# Determinants of water source use, quality of water, sanitation and hygiene perceptions among urban households in North-West Ethiopia: A cross-sectional study

**DOI:** 10.1371/journal.pone.0239502

**Published:** 2021-04-22

**Authors:** Shewayiref Geremew Gebremichael, Emebet Yismaw, Belete Dejen Tsegaw, Adeladilew Dires Shibeshi

**Affiliations:** 1 Department of Statistics, Debre Tabor University, Debre Tabor, Ethiopia; 2 Department of Mathematics, Debre Tabor University, Debre Tabor, Ethiopia; Kaohsiung Medical University, TAIWAN

## Abstract

**Background:**

Clean water is an essential part of human healthy life and wellbeing. More recently, rapid population growth, high illiteracy rate, lack of sustainable development, and climate change; faces a global challenge in developing countries. The discontinuity of drinking water supply forces households either to use unsafe water storage materials or to use water from unsafe sources. The present study aimed to identify the determinants of water source types, use, quality of water, and sanitation perception of physical parameters among urban households in North-West Ethiopia.

**Methods:**

A community-based cross-sectional study was conducted among households from February to March 2019. An interview-based a pre-tested and structured questionnaire was used to collect the data. Data collection samples were selected randomly and proportional to each of the kebeles’ households. MS Excel and R Version 3.6.2 were used to enter and analyze the data; respectively. Descriptive statistics using frequencies and percentages were used to explain the sample data concerning the predictor variable. Both bivariate and multivariate logistic regressions were used to assess the association between independent and response variables.

**Results:**

Four hundred eighteen (418) households have participated. Based on the study undertaken,78.95% of households used improved and 21.05% of households used unimproved drinking water sources. Households drinking water sources were significantly associated with the age of the participant (x^2^ = 20.392, df = 3), educational status (x^2^ = 19.358, df = 4), source of income (x^2^ = 21.777, df = 3), monthly income (x^2^ = 13.322, df = 3), availability of additional facilities (x^2^ = 98.144, df = 7), cleanness status (x^2^ = 42.979, df = 4), scarcity of water (x^2^ = 5.1388, df = 1) and family size (x^2^ = 9.934, df = 2). The logistic regression analysis also indicated that those factors are significantly determining the water source types used by the households. Factors such as availability of toilet facility, household member type, and sex of the head of the household were not significantly associated with drinking water sources.

**Conclusion:**

The uses of drinking water from improved sources were determined by different demographic, socio-economic, sanitation, and hygiene-related factors. Therefore; the local, regional, and national governments and other supporting organizations shall improve the accessibility and adequacy of drinking water from improved sources in the area.

## 1. Background

Clean water is an essential element for human health, wellbeing, and prosperity [1]. Every human being has the right to access safe drinking water. Currently, about one billion people, who live in the developing world, don’t have access to safe and adequate drinking water [2]. Water can be found from either improved or unimproved water sources. Improved sources include piped supplies (such as households with tap water in their dwelling, yard, or plot; or public stand posts) and non-piped supplies (such as boreholes, protected wells and springs, rainwater, and packaged or delivered water) [3,4]. To the contrary, unimproved water sources provide water collected from unprotected dug wells, unprotected springs, and surface water. The opposite of improved water sources has been termed unimproved water sources, based on the Joint Monitoring Program (JMP) [5] definitions.

Globally, between 2000 and 2015, the population using piped supplies increased from 3.5billion to 4.7 billion, while the population using non-piped supplies increased from 1.7billion to 2.1 billion. Evidence shows that globally two out of five people in rural areas and four out of five people in urban areas use piped supplies [4]. About 748 million people, mostly the poor and marginalized, there is a scarcity of using an improved water source supply and of these, almost a quarter (173 million) rely on untreated surface water, and over 90% live in rural areas [6]. About 547 million people did not have an improved drinking water supply in 2015. In a study conducted by Water.org [7], 42% of the population has access to a clean water supply and only 11% of that number has access to adequate sanitation services globally.

In Africa, only 60% of the population has access to improved sanitation services, but the situation is worse in rural areas, in which below half (45%) of the rural population has access to improved sanitation services. According to the World Health Organization (WHO), 2011 report, individuals with no access to improved sanitation are forced to defecate in open fields, in rivers, or near areas where children play and food is prepared [8].

The Ethiopian Demographic and Health Survey (EDHS) (CSA and ICF, 2016) [9] reported that 97% of urban households in Ethiopia have access to an improved source of drinking water and in rural areas, only 57% have improved water accessibility. Nevertheless, no reliable information is available on the readability of drinking water quality reports for further illustration [10]. Based on previous reports, Ethiopia is the country with the worst of all water quality problems in the world. It has the lowest water supply (42%) and sanitation coverage (28%) in sub-Saharan countries [11]. Ethiopia is considered as having one of the poorest sanitation and drinking water infrastructures [12]. About 52.1% of the population has been using unimproved sanitation facilities while 36% of them practiced open defecation [13]. In Ethiopia the discontinuity of drinking water supply affects the distribution of water to the community in need [14].

Due to the lack of accessibility of water in many rural areas, females are put to work on collecting water each morning to help their families [15,16]. The burden of water collection does not fall equally on all household members; the gender breakdown is consistent for both urban and rural areas [17]. The responsibility of collecting water-primarily falls on women, sons, or daughters of the households. Globally; women (64%), men (24%), girls (8%), and boys (4%) share the burden of collecting water [18]. Due to the presence of a burden on children, only 45% of kids attend primary education in Ethiopia [15], and also, water and sanitation-related sicknesses put severe burdens on health services and keep children out of school [19]. Younger household members are more likely to collect water, but this differs by place of residence; while only 22 percent of those who collect water in urban areas are children (aged 7 to 14). In rural areas, nearly 37 percent of water collectors are children [20].

The discontinuity of drinking water supply forces households to use water storage material or to use water from unimproved sources. In our study area, irregularity of water supply was observed and the community is forced to use unprotected water storage materials. Water stored in unprotected materials (such as unsafe Pot, Rotto, Jerikan, other plastic materials) for longer periods of might get contaminated and cause water-borne diseases. The water from unimproved water sources might be contaminated with animals, floods, and specks of dust through wind and human wastes. This ultimately causes human sickness.

As a result of different reasons, there is a continuing dearth of information on the identification of determining factors of using improved sources of drinking water in the study area. We aimed to address this knowledge gap and explored in detail, access, usage, and practices of water sources in North-West Ethiopia. This also provides valuable insights into access to safe water and consequentsocioeconomic conditions such as income, distances, and family size that can become a barrier to water access.

## 2. Methods

### 2.1 Study design, area and period

**Fig 1:**
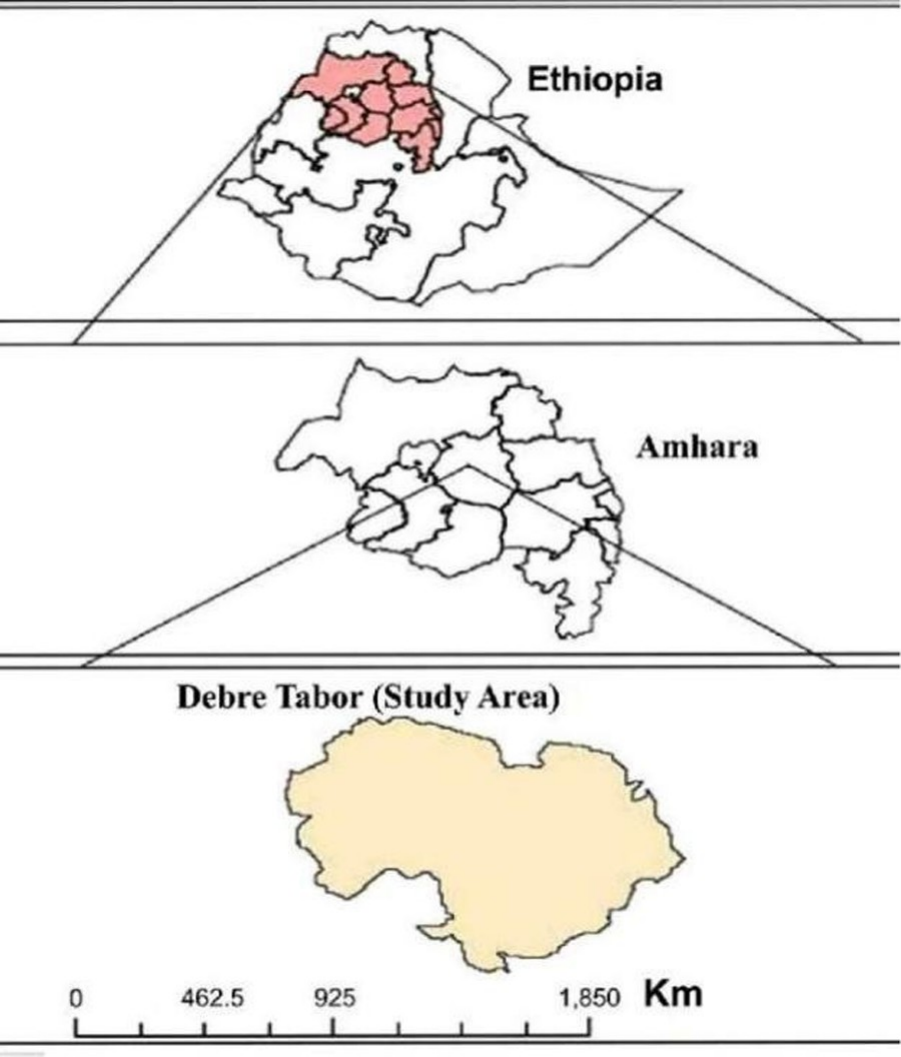
Location map of the study area.

A community-based cross-sectional study design was conducted from February to March2019. The study was conducted in Debre Tabor District, which is situated in North-West Ethiopia (Fig 1). Based on the district official report, the population of the town is expected to be 87,627 (2019 projected population). From this population a number of 49,535 households members are users of tap water in 2019, but the remaining 38,092 household members are not tapped, water users. Drinking water of the town comes from large reservoirs located in its surroundings, Farta Woreda, which is one of the administrative Woredas in the South Gondar Zonal Administration. In the district, there are a lot of wells, which were extracted to supplement the domestic water requirement in town. Based on previous evidence, the households of Debre Tabor town get pipe water supply only once a week [21].

### 2.2 Sample size determination

The town has about 17,526 households. The studied part of the population is made up of families that reside in the town, which comprise of men, women, and children, all of which are in different age groups. The average family size of the town was computed asabout4.53 per household (after a pilot survey was conducted in January 2019).

For the household survey, samples were selected using the sample size determination equation of Cochran (1977) [22]. The study used a single proportion formula, 95% confidence interval, the marginal error of 5%, and the non-response rate of 10%;

$$n=\frac{Z^{2}(1-P)}{d^{2}(N-1)+Z^{2}P(1-P)}$$

Where;

Z = 95% confidence limit (z-value at α = 0.05 is 1.96); N = Number of households in Debre Tabor town = 17526; p = 0.5; 1-p = 0.5; D = Marginal error or degree of accuracy = 0.05; n = 380+38 = 418.

Total sample size = 418. However, a sample size of 418 was used to eliminate any errors. The town has 6 kebeles (wards). The samples were selected randomly and taken proportionally from each kebeles and sub-kebeles.

### 2.3 Sampling method and sampling procedure

All households living in the study area at the time of data collection were included as the study population. Data collection sites were demarcated into 6 kebeles. The samples were selected randomly using a proportional allocation from each kebeles (wards) and sub-kebeles (sub-wards).

### 2.4 Data collection tools and techniques

The primary source of data was employed. The primary data gathering was including, household survey questionnaires (on paper) and personal observation. The content of the questionnaire was checked by public health professionals, who have had a profound experience in the area. The method of data collection was by investigator administered questionnaire. The investigators/researchers (people who speak the Amharic language) administered a questionnaire to participants regardless of their educational level. Seven data collectors were selected for data collection and two supervisors were assigned.

The questionnaire consisted of five sections namely; section I: socio-demographic data, section II: Sources of income, section III: water source observation, section IV: household water use and section V: Water quality and sanitation perception. There were key informant interviews and verification of the facilities using a checklist.

A detailed questionnaire was prepared in the native language of the households (Amharic) and included over 50 questions. A multiple-choice format was used to answer the majority of the questions. House-hold characteristics, such as the number of family size, educational level, monthly income of the household, type of occupation, sources of water, sanitation and hygiene, and awareness about household drinking water were included.

### 2.5 Study variables

The variables included in this study were taken based on perceptions of households and not verifiable water quality measures.

#### 2.5.1 Dependent variables

The dependent variable was the source of drinking water (improved, unimproved). Based on WHO guidelines improved water sources consisted of piped water into dwellings, yards/plots, and public taps/stand-pipes, tube-wells/bore-holes, protected wells, protected springs, and rainwater. Bottled water was included as an improved water source if the household used another improved water source for other purposes, such as hand-washing and cooking. Unprotected wells, unprotected springs, tankers, trucks, a cart with tank/drum, and surface water were considered as ‘unimproved water sources’.

#### 2.5.2 Independent variables

The independent variables included in this study are demographic, socio-economic, sanitation, and hygiene perception characteristics.

- The perceptions of households using water from unimproved sources(income, distance from home to water source, the presence of alternative water source, quality of water perception, adequacy of water, waiting time to fetch water, personal interest, and other reasons).
- The presence of scarcity of water in the area (yes, no).
- The reason that households believe the presence of the scarcity of water has occurred (government weakness, a local people problem, and both local people and government problems).
- The perception of households of the water they consume has a safety status (not safe at all, somewhat safe, partially safe, safe, and highly safe).
- Households’ perception of the indicator of water quality (color, taste, odor, disease attack, and the presence of all the cases).
- Households’ perception of the taste, odor, and color of the water from the improved and unimproved sources was the same (yes, no).
- The causes of water quality problem households perceive (water-containing material, animal wastes, human wastes, flood, and all cases).
- Treatment measures households had undertaken during unsafe drinking water (no use at all, boiling, sedimentation, using wuha agar, other methods, and use all measures).
- The number of times household members had got sickness due to water-related disease and visited health centers for physician assistance within one year before the survey time (not at all, once, twice, three times, more than three times).
- The presence of health extension workers’ assistance (yes, no) and the number of times the family was visited by health extension workers within one year before the survey (not at all, once, twice, three times, more than three times).
- Previous participation of household members in educational and awareness activities about sanitation and hygiene in their locality (yes, no). The presence of a latrine facility in the household compound (yes, no) and who have used the latrine (wife, husband, children, and all families, except children).The place household members were defecating (public, neighbor, open place, own toilet), and the presence of the culture of households washing hands after defecation (yes, no).

### 2.6 Data quality assurance

A pilot survey (pre-test) was incorporated to recheck the questionnaire and for sample size determination. Before the main survey, a mini-survey (pilot survey) was done on 30 households outside the study area (Bahir Dar city) to avoid exclusion of households who were in the study area due to the pre-test. After the pre-test questionnaire was done; question order, alternative option, skip pattern and an overlapping option were amended. Supervisors and data collectors were trained by the principal investigator. During data collection, data collectors were supervised by supervisors in close up.

### 2.7 Operational definitions

- Awareness: Understanding the implication and becoming conscious of conditions and practices concerning many things including hygiene and health.
- Improved sanitation: A sanitation system that is connected to the public sewer, septic tank and a pours toilet/latrine. For a simple pit latrine, it implies the use of a slab and ventilated improved latrine.
- Safe water: A water system that is well protected from contamination sources, treated with chemicals, and used in ways that prevent contamination.
- Sanitation: Act of cleanliness and containment of waste products to make the living and working environment free from matters that affect health and wellbeing.

### 2.8 Data processing and analysis

The collected data were coded and entered into MS Excel, cleaned, stored, and exported into R version 3.6.2 for analysis. Any error that occurred during data entry was corrected by revising the originally completed questionnaire. Descriptive statistics using frequencies and percentages were used to explain the sample data concerning the predictor variable. Both bivariable and multivariable logistic regression estimates were used to assess the association between the independent and the response variables. During the bivariate analysis p-value of 0.2 was included in the multivariate analysis to control the association of confounding variables with that of the response variable. In the multivariable analysis of binary logistic regression, variables with a p-value of 0.05 and 95% confidence interval were considered as statistically significant.

### 2.9 Ethical considerations

Prior to the study an ethical permission was obtained from Debre Tabor University, Faculty of Natural and Computational Sciences institutional review board (IRB). Verbal consent was obtained from study participants. Since the study participants were aged 18 and above years, we have not obtained written informed consent from participants. The confidentiality and privacy of participants were actively protected. All participants were assigned a unique identification number. Every effort was made to emphasize the volunteers of this study and decisions to stop prior to the interview were made.

## 3. Results

The summary statistics for different explanatory variables (Table 1) and the cross- tabulations among the types of water sources used by the households and the demographic, economic, sanitation, and hygiene perception of households (Table 2) are presented. The results of chi-square tests of association and logistic regression are presented (Table 2).

**Table 1:** Summary statistics of water source using practice, quality and sanitation perception among urban households in North-West Ethiopia, 2019.

**Why household use unimproved source of water?**		
	Frequency	Percentages (%)
Income	51	12.20
Distance	19	4.55
Presence of alternative sources	19	4.55
Quality	83	19.86
Adequacy	15	3.59
Waiting time	7	1.67
Interest	20	4.78
Others	200	47.84
All cases	4	0.96
Total	418	100
**Presence of scarcity of water source in the area**		
Yes	381	91.15
No	35	8.37
No answer	2	0.48
Total	418	100
**If scarcity of water, the reason they perceive is due to:**		
Government	358	85.65
Local people	49	11.72
Both (government and local people	9	2.15
No answer	2	0.48
Total	418	100
**Water consumed safety status perception by households**		
Not safe at all	2	0.48
Somewhat safe	23	5.50
Partially safe	82	19.62
safe	228	54.55
Highly safe	83	19.85
Total	418	100
**Indicator of water quality**		
Color	129	30.86
Taste	75	17.94
Odor	61	14.59
Disease attack	16	3.83
All cases	121	28.95
Cannot be determined	16	3.83
Total	418	100
**Is taste, odor and color of water from unimproved source same as improved source?**		
Yes	107	25.60
No	305	72.96
Cannot be determined	6	1.44
Total	418	100
**Cause of water quality problem**		
Water containing material	219	52.39
Animal wastes	71	16.99
Human wastes	47	11.24
Flood	27	6.46
All causes	54	12.92
Total	418	100
**Treatment measure for unsafe drinking water**		
Not at all	36	8.61
Boiling	286	68.42
Sedimentation	73	17.46
Using chemical treatment	2	0.49
Others	8	1.91
All measures	13	3.11
Total	418	100
**Number of times family sick due to water related disease in the previous 1 year**		
Not at all	353	84.45
Once	6	1.44
Twice	51	12.20
Three times	6	1.44
More than three times	2	0.48
Total	418	100
**If your family sick due to water related disease, how many times you visited healthcenters?**		
Not at all	353	84.45
Once	6	1.44
Twice	37	8.85
Three times	20	4.78
More than three times	2	0.48
Total	418	100
**Visited by Health Extension Workers (HEWs) in previous one year**		
Yes	168	40.19
No	250	59.81
Total	418	100
**If you have visited by HEWs, how many times it was?**		
Once	103	61.31
Twice	51	30.36
Three times	9	5.36
More than three times	5	2.97
Total	168	100
**Participation in Educational and Awareness Activities about Sanitation and Hygiene**		
Yes	77	18.42
No	341	81.58
Total	418	100
**Availability of latrine**		
Yes	408	97.61
No	10	2.39
Total	418	100
**Who use the latrine**		
Husband	0	0
Wife	0	0
Children	0	0
All families	402	98.53
Except children	6	1.47
Total	408	100
**Where do you defecate?**		
Public toilet	4	0.96
Neighbor toilet	2	0.48
Open field	15	3.59
Own toilet	397	94.97
Total	418	100
**Washing hand after defecation**		
Yes	374	89.47
No	44	10.53
Total	418	100

(Source: Survey February 2019).

**Table 2:** Determinant factors of sources of drinking water of households.

Exploratory Variables	Source of Drinking Water(SDW)		Binary Logistic Regression Result		
	Improved	Unimproved	AOR	95% CI for AOR	P-value
**Availability of toilet facilities**					
No	6(1.82%)	4(4.50%)	1	[0.0021, 1.1600]	0.066
Yes	324(98.18%)	84(95.50%)	0.053		
X^2^ = 1.251*, df = 1, p-value = 0.2634					
**Household member type**					
**Child**	20(6.06%)	2(2.27%)	**1**	[1.1537,8.2490]	**0.990**
**Spouse**	310(93.94%)	2(2.27%)	**2.777**		
X^2^ = 1.258*, df = 1, p-value = 0.262					
**Sex of household head**					
**Female**	120(36.36%)	38(43.18%)	**1**	[0.0324,1.6494]	**0.449**
**Male**	210(63.64%)	50(56.82%)	**0.073**		
X^2^ = 0.807*, df = 1, p-value = 0.3691					
**Age of household head(years)**					
<18	1(0.30%)	2(2.27%)	**1**	-	-
**18–30**	69(20.91%)	2(2.27%)	**6.171**	[1.639,9.313]	0.019 *
**31–45**	174(52.73%)	54(61.36%)	**6.900**	[1.601,9.887]	0.048 *
**>45**	86(26.06%)	30(34.00%)	3.244	[2.003,8.440]	0.041 *
X^2^ = 20.392, df = 3, p-value = 0.0001					
**Educational Background**					
Illiterate	34(10.30%)	15(17.05%)	**1**	-	-
Read and write	44(13.34%)	10(11.36%)	0.121	[0.0171,0.0721]	0.025 *
High school complete	40(12.12%)	24(27.27%)	4.407	[1.0578,21.3510]	0.051.
Diploma Complete	94(28.48%)	15(17.05%)	0.434	[0.1351,1.322]	0.150
Degree and above complete	118(35.76%)	24(27.27%)	0.015	[0.0148,1.2372]	0.091.
X^2^ = 19.358, df = 4, p-value = 0.0007					
**Source of income**					
Agriculture	6(1.82%)	10(11.36%)	**1**	-	-
Government employer	193(58.48%)	42(47.73%)	1.862	[1.0647,11.5460]	0.049 *
Merchant	58(17.58%)	23(26.14%)	1.0531	[1.0529,5.1995]	0.062.
Self-employed	73(22.12%)	13(14.77%)	1.0182	[1.0085,1.5287]	0.045 *
X^2^ = 21.777, df = 3, p-value = 0.001					
**Monthly income(in birr)**					
Below 1500	49(14.85%)	18(20.45%)	1	-	-
1501–3000	81(24.54%)	34(38.64%)	2.228	[0.4812,10.779]	0.305
3001–5000	162(49.09%)	34(38.64%)	1.990	[1.0850,2.545]	0.001 **
Above 5001	38(11.52%)	2(2.27%)	1.390	[1.0034,2.102]	0.002 **
X^2^ = 13.322, df = 3, p-value = 0.004					
**Facilities observed in the area**					
Not at all	109(33.03%)	2(2.27%)	1	-	-
Cattle trough	10(3.03%)	19(21.59%)	1.553	[0.120,20.086]	0.994
Showers	38(11.52%)	0(0.00%)	0.227	[0.018,2.942]	0.992
Washing dish	126(38.18%)	48(54.55%)	0.032	[0.002,0.432]	0.044 *
Fences	37(11.21%)	5(5.68%)	0.067	[0.005,0.908]	0.034 *
Washing dish/fences	4(1.21%)	2(2.27%)	0.201	[0.062,0.656]	0.0064 **
Washing dish/shower	6(1.82%)	8(9.09%)	17.322	[0.952,31.072]	0.994
All	0(0.00%)	4(4.55%)	4.734	[2.383,8.033]	0.005 **
X^2^ = 98.144, df = 7, p-value = 0:001					
**Water scarcity**					
No	34(10.30%)	2(2.27%)	1	-	-
Yes	296(89.70%)	86(97.73%)	6.178	[2.788,12.854]	0.067.
X^2^ = 5.1388*, df = 1, p-value = 0.0234					
**Cleanness status**					
Not clean	2(0.61%)	6(6.82%)	1	-	-
Partially clean	71(21.52%)	32(36.36%)	1.184	[0.152,13.242]	0.423
Somewhat clean	14(4.24%)	12(13.64%)	3.494	[1.597,7.390]	0.024 *
Clean	210(63.64%)	38(43.18%)	3.920	[2.316,5.977]	0.000 ***
Very clean	33(10.00%)	0(0.00%)	4.860	[0.944,3.782]	0.991
X^2^ = 42.979, df = 4, p-value = 0.001					
**Family size**					
< = 2	20(6.06%)	5(5.68%)	1	-	-
3–5	243(73.64%)	78(88.64%)	0.694	[0.592,0.814]	0.049 *
6	67(20.30%)	5(5.68%)	3.421	[2.312,5.063]	0.099.
X^2^ = 9.934, df = 2, p-value = 0.007					

**NB**: 1: Reference Category, *Yates’continuity correction (for X2 values) Significant codes: 0‘***’ 0.001 ‘**’ 0.01 ‘*’ 0.05 ‘.’ 0.1 ‘‘ 1.

### 3.1 Summary statistics of explanatory variables

The summary statistics of the different explanatory variables used in this study are presented in (Table 1).

### 3.2 Unimproved water source using practice

Households use both improved and unimproved water sources for their daily water consumption. Based on the present survey, about 330(78.95%) and 88(21.05%) households used improved and unimproved sources of water; respectively. Even if an improved source of water is of good quality, it is not readily available. Sources of water (improved, unimproved) versus different explanatory variables were presented (Table 2). The reason why households respond to using unimproved sources of water thanimproved sources of water were shown (Table 1).

Households using unimproved sources of water are due to income 51(12.20%), distance 19(4.55%), presence of alternative sources 19(4.55%), quality 83(19.86%), adequacy 15(3.59%), waiting for time 7(1.67%), interest 20(4.78%), all cases 4(0.96%), and other (cases other than the listed) 200(47.84%) than the improved sources of water. The quality of the improved sources of water is indeed better than the unimproved sources of water. About 83(19.86%) households preferred unimproved sources of water to improved sources. This might be due to the accessibility of unimproved sources. Nearly half or 200(47.84%) households preferred unimproved sources other than the reasons of income, distance, presence of alternative source, quality, adequacy, waiting time, and interest. Future investigations to determine the factors (other than listed in this study) that households prefer unimproved sources than improved sources shall be undertaken.

Of 418 household respondents, about 381(91.15%) perceive that water is scarce in the area; while 35(8.37%) respondents perceive that there is no scarcity of water. Two (0.48%) respondents refused to answer about the presence or absence of the scarcity of water in the area. From this, we can summarize that the scarcity of water is a serious issue because above 90% of households perceive that they live under scarce conditions. For about 200(47.84%) households, the reason to use unimproved sources were "Others" (i.e., other than the listed). This might be due to the presence of scarcity of water from improved sources that households would like to use from unimproved sources.

If water was scarce, the respondents’ households asked about who would be responsible for it. Of 418 households 358(85.65%) due to government, 49(11.72%) due to local people, 9(2.15%) due to both government and local people perceive that water scarcity occurred. About 2(0.48%) households who refused to say about the presence or absence of the scarcity of water didn’t like to state the concerned body to be responsible for.

### 3.3 Water safety status and quality indicator perception

The water safety status was presented by the Likert scale from low safety status to high safety status. The summary statistics (Table 1) show the water-consuming safety status of 418 respondent households. About 83(19.85%) responded that the water consumed is highly safe, 228(54.55%) safe, 82(19.62%) partially safe, 23(5.50%) somewhat safe, and 2(0.48%) not safe at all; they believed about the water they consumed. For the majority of about 311(74.40%) households, the water they consumed was safe and highly safe. Even if for the majority (three-fourths) of the respondents the water safety status was safe; the remaining households were targeted to improve their water safeness.

From 418 responding households about 402(96.17%) had different perceptions about the quality of water being consumed from different sources. About 129(30.86%) made complaints about the color, 75(17.94%) said it hadbad taste, 16(3.83%) assumed microbial contamination, 61(14.59%) complained about its odor, for 121(28.95%) all above cases were provided, while about 16(3.83%) can’t determine the water quality which they consumed daily.

To know the general knowledge about the quality of water from improved and unimproved water sources, a question was posed to the households: "Is taste, odor, and color of the water from the unimproved source the same as from the improved source?" Of 418 respondents 107(25.60%) responded with "yes", about 305(72.96%) answered "no", while about 6(1.44%) responded that they cannot determine. The majority of 305(72.96%) households could not differentiate the quality of water using taste, odor, and color, either from improved or unimproved water sources. Even microorganisms cannot be identified by taste, odor, and color easily; using those identifiers to differentiate the water quality as cheap and fast in-door activity.

There are different causes of water quality problems from the source and in-door in accordance with the household reports. In-door, the water storage material takes the higher cause of the water quality problem. From the source animal and human wastes and floods are the main causes. (Table 1) provides the different causes of water quality problems for the 418 households:219(52.39%) claim due to water containing material, 71(16.99%) due to animal wastes, 47(11.24%) due to human wastes, and27(6.46%) due to flood, and 54(12.92%) due to all mentioned cases.

Of 418 households about 286(68.42%) used boiling, about 73(17.46%) used sedimentation, about 2(0.49%) used chemical reagent, about 8(1.91%) used other treatment measures, about 13(3.11%) used all treatment measures (boiling, sedimentation, chemical); alternatively, while about 36(8.61%) didn’t use any treatment measures for unsafe drinking water. The majority of 286(68.42%) households’ used boiling treatment measure is due to its undergone in-door, cheap, and easy. Modern treatment measures like using chemical reagents are not common and accessible for low-income households. There are individuals who don’t use any measure to treat unsafe drinking water.

From 418 households, about 353(84.45%) household families were not sick at all due to water-related diseases in the previous 1 year. About 6(1.44%) household families were getting sick once due to water-related diseases and they had visited health centers; about 51(12.20%) household families were sick twice due to water-related diseases and of those about 37(8.85%) had visited health centers; about 6(1.44%) household families were sick three times and all had visited health centers, about 2(0.48%) household families were sick four and more times and all had visited health centers. Households, who were sick twice per year, had visited health centers three times. At the time, when the disease had gone worse, individuals visited health centers repeatedly. It is unusual for families who aren’t sick due to water-related diseases to visit health centers (i.e., all 353(84.45%) household families’, which werenot sick due to water-related diseases hadnot visited health centers for water-related diseases).

The status of households, who were visited by health extension workers (HEWs) per year are shown in Table 1.Of 418 households about 168(40.19%) were visited by HEWs, while 250(59.81%) households were visited by HEWs in the previous year. Of 168(40.19%) households, who had visited by HEWs, 103(61.31%) visited once, 51(30.36%) visited twice, 9(5.36%) visited three times and 5(2.97%) households had visited four and more times per year. More than half of the responded households were never visited by HEWs. It shows poor management of the town health bureau. In the places where the communities live densely, communities needed assistance for good health and quality of life to eradicate communicable diseases. Health extension workers (HEWs) played a great role in the development of community health and quality of life. HEWs participated in the quality of life and family planning in the previous 15 years in the country.

### 3.4 Sanitation and hygiene practice

The participation of household members in educational and awareness activities about sanitation and hygiene was playing a great role in a healthy community and a clean environment. Of 418 households included in the survey, about 77(18.42%) had participated; while about 341(81.58%) had not participated in educational and awareness activities concerning sanitation and hygiene in their locality. Community education and awareness activities about good health, quality of life (QoL), sanitation, and hygiene were provided by health extension workers (HEWs) in the area. But, in the town, only 168(40.19%) households were visited by HEWs.

Regarding the availability of latrines in the households, about 408(97.61%) households had a latrine in the compound and/or surrounding for defecation; while about 10(2.39%) households did not have a latrine. Of those who had a latrine 408(97.61%) households), only about 402(98.53%) household toilets were accessible to all families. However, about 6(1.47%) household toilets were accessible for families except for children. About 397(94.97%) used their own toilet, about 15(3.59%) used open-field, about 4(0.96%) used public toilet, and about 2(0.48%) used the neighbor’s toilet for defecation. Open field toilet defecation was found within dense trees and drainage areas, which could easily disturb the surrounding environment and be eroded by the flood. Households use neighbor’s toilets at a time when their home and/or safety tank were at a building stage.

The culture of household members washing their hands after defecation, of 418 households about 374(89.47%) households had washed their hand, while about 44(10.53%) households hadn’t adopted the culture of washing hands after defecation. Among 374(89.47%) households had washed their hand with water and additionally with soap, but were inconsistently using soap. The country also motivated hand washing after defecation by memorizing a day per year, nationally.

### 3.5 Predictors of access to improved sources of drinking water

The chi-square test of association and the binary logistic regression results about the predictors of access to improved sources of drinking water were shown (Table 2). The results demonstrated that there was no significant association between the availability of toilet facilities and types of water sources used by residents of the town (x2 = 1.251, df = 1, p-value = 0.2634). The availability of toilet facilities with improved sources was 98.18% (OR = 0.053; 95%CI: 0.0021–1.1600).

There was no significant association between household member type (x2 = 1.258, df = 1, p- value = 0.262) and the sex of the household head (x2 = 0.807, df = 1, p-value = 0.3691) with the type of water sources used by residents of the town. Household member type being spouse was 93.94% (OR = 2.78; 95%CI: 1.1537–8.2490) to be accessible to improved sources of drinking water. The sex of the household head being male was 63.64% (OR = 0.073; 95%CI: 0.0324–1.6494) to be accessible to improved sources of drinking water. Based on the 95% CI for the odds ratio of both household member type and sex of the household head did not significantly determine the source of water used by households.

There was a significant association between the age of the household head and the type of water source used by residents of the town (x2 = 20.392, df = 3, p-value = 0.0001). The age of the household head (18–30 years) was 6.171 times higher than the age of being below 18 years; with 20:91% (95%CI: 1.639–9.313; p-value = 0.019). The accessibility of improved sources of drinking water was lower in the older age group (> 45 years) than the medium age group (18–30 and 31–45 years), but better than the younger (below 18 years).

The educational status of household heads was significantly determined by the presence of improved sources of drinking water within the households (x2 = 19.358, df = 4, p- value = 0.0007). Being able to read and write 0.121 times 13.34% (95%CI: 0.0171–0.0721), diploma complete 0.434 times 28.48% (95%CI: 0.1351–1.322); and degree and above complete 0.015 times 35.76% (95%CI: 0.0148–1.2372) were lower than illiterate household heads to be accessible to improved sources of drinking water. However, household heads with high school complete were 4.407 times 12.12% (95%CI: 1.0578–21.3510) higher than illiterate household heads.

There was a significant association between the main source of income of the households and the type of water sources used by the town residents (x2 = 21.777, df = 3, p-value = 0.001). The main source of income of households’ through self-employer 22.12% (OR = 1.0182; 95%CI: 1.0082–1.5287), merchant 17.58% (OR = 1.0531; 95%CI: 1.0529–5.1995), and government employer 58.48% (OR = 1.862; 95%CI: 1.0647–11.5460) significantly determined the source of water from improved sources compared to households’ whose source of income was through agriculture. The accessibility of improved sources of drinking water was 86.2% increased for government-employed compared to agriculture income households.

Additionally, there was a significant association between the monthly income of households and the source of water used by the town residents (x2 = 13.322, df = 3, p-value = 0.004). Monthly income (1501–3000)(birr) was 24.54%(OR = 2.228; 95%CI:0.4812–10.779);income (3001–5000)(birr) was 49.09%(OR = 1.990; 95%CI: 1.0850–2.545); and income above 5001 (birr) 11.52%(OR = 1.39; 95%CI: 1.0034–2.102) compared to lower than 1500 (birr) income households. Facilities such as:—washing dish 38.18% (OR = 0.032; 95%CI: 0.002–0.432); fences 11.21% (OR = 0.067; 95%CI: 0.005–0.908), both washing dish and fences 1.21% (OR = 0.201; 95%CI: 0.062–0.656), and all facilities (OR = 4.734; 95%CI: 2.383–8.033) compared to no facilities at all observed to determine the source of water from improved sources, but not cattle trough and showers (x2 = 98.144, df = 7,p- value = 0.001).

There was a significant association between the cleanness status of the surrounding and the type of water used by the town residents (x2 = 42.979, df = 4, p-value = 0.001). Somewhat clean 4.24% (OR = 3.494, 95%CI: 1.597–7.390) and to be clean 63.64% (OR = 3.92, 95%CI: 2.316–5.977) compared to no clean surrounding were observed to determine the source of water from improved sources, but not partially clean and very clean surrounding cleanness status categories.

About two-hundred ninety-six (89.70%) of households had a scarcity of water from improved sources. Nearly nine out of ten households were under a scarcity of water from improved sources. 6.178 times (odds) of water scarcity occurred from improved sources than water from non-improved sources (95%CI: 2.788 12:854) and (x2 = 5.1388, df = 1, p- value = 0.0234).

Households with large family sizes had access to water from improved sources. Family size (3–5) was73.64% (OR = 0.694; 95%CI: 0.592–0.814), and family size (> = 6) was 20.30% (OR = 3.421; 95%CI: 2.312–5.063) compared to lower number of family sizes (< = 2). From this, we can conclude that a medium number of family size (3–5) got lower odds (decreased by 69.4%) to improve sources but a higher number of family size (> = 6) had gained higher odds (3.42 times) to improved sources.

## 4. Discussion

Different studies showed that many factors affect the supply of quality drinking water in households such as the age of household members [23–25], the age of the household head [26], the gender of household members [23,27–35], occupation of the household head [36], improved and unimproved water sources in rural and urban areas [23,27,37–39], households standard of living (income) [40–46], education level of household members [34,47], household size and composition [27,48–51]. The present study shows the associated factors of drinking water sources in urban households.

A study was undertaken in the area also concludes that nine of ten persons were under the problem of water scarcity; the supply was inadequate, and the quality was low [52]. The current study found that about 78.95% of the town population was using water from improved sources, while about 21.05% were using water from unimproved sources. A study was undertaken in the surrounding rural areas of the Debre Tabor Town, Farta district showing that about 57.10% of the population had access to improved water sources and the remaining from unimproved sources [53].

### Unimproved water source using practice

The population of the town used both improved and unimproved water sources for their daily consumption. Households use unimproved sources of water that were associated with several reasons such as- income, distance, presence of alternative sources, quality, adequacy, waiting for time, interest, and other cases. About 4.55% of the population due to distance and 19.86% of the population due to quality used unimproved sources. Improved water sources in urban areas were located in short distance [54], and the quality of water was better from improved sources.

About 91.15% of the town population was under the problem of drinking water scarcity. It indicates that the supply was below 10%. The figure was lower compared to 60% of the population that had access to improved water sources in Africa and 42% water supply in sub-Saharan African countries [55]. About one billion population in the world has no access to safe and adequate water sources [56].The country report shows the presence of poor sanitation and drinking water infrastructure [12]. The lower supply of drinking water and the unimproved sanitation of households are associated [13]. It also affects the distribution of water in the area [14], and it leads to health risks [57,58]. The EDHS report in 2016 [59] indicates that 97% of the urban population in Ethiopia had access to an improved source of drinking water, even if its quality was not clear [60]. However, in the current setting, the supply was below 10%. A report [15] reasons out that the problem was occurring due to drought and the Horn of Africa regional instability. An international report of [20] also suggested the push and pool factors of poor water and sanitation.

In the study area, 85.65% of the population perceived that the scarcity of water was associated with poor administration factors of the local, regional, and national government. This might coincide with the international report [20] of different factors, such as the absence of good drinking water infrastructure [12] and discontinuous supply of drinking water [14]. In most African countries water scarcity was more severe in rural localities than in urban settings [61].

### Water safety, quality and sanitation perception

The presence of drinking water is vital for every human being. About 74.40% of the population in the study area consumes safe water, but about a quarter of the population consumes this below the standard of safety. There is no pure water in nature [62], and about a billion people in the world don’t have access to safe drinking water [56]. Since unsafe water leads to water-borne diseases [41,63], it is specially at higher risk in rural households [64].The local government shall take this mandate to balance the right and responsibilities of its people. Water-borne diseases are a major concern for households [36] and highly affect households from developing countries who live in extreme conditions of poverty [65]. The risk of lack of safe water is more than from any man-made destruction (such as war, terrorism, and toxic weapons) [66]. Globally, millions of people die as a result of water-related diseases following the WHO report [66].

Households use different perceptions to identify the quality of water they consume daily. They have used color, taste, possible contamination with infectious diseases, and odor. About 3.83% of the study population couldn’t determine the water quality they consumed. Households have got the water they consumed from improved and unimproved sources.”Are the water perceptions similar from those two sources?" was asked to the households. About 25.60% of the population responded with similar perceptions were observed, but about 1.44% of the population responded as they couldn’t determine. About 72.96% of the population couldn’t differentiate the quality of water using taste, odor, and color, either from improved or unimproved sources.

In the developing world, peoples commonly do not have access to safe water [56] and some defecate in an open field [8]. Water sources are contaminated with domestic and industrial wastes [12,66]. About half of the study population was subjected to poor water quality due to water containing material, poor in-door practices; 16.99% were due to animal waste, and 11.24% due to human waste. In a place where water shortage is available, water may get stored for a long time. Water stored from 1 to 9 days increased the contamination level by 67% [57]. In the current setting as investigators observed, households stored water for several days. A study also shows that the town population had got water once per week [21]. The presence of animal and human wastes resulted in poor sanitation and hygiene. This leads to water-related sickness and diseases [41,58,65,66].

When unsafe drinking water was observed, households used treatment measures such as boiling, sedimentation, and chemicals, and a combination of two or more. 68.42% of the study population used boiling as a means of treatment measure, and 17.46% were using sedimentation. This might be due to the lower cost. Unsafe drinking water is the cause of many water-borne diseases and leads to health disorder.

About 84.45% of the study population didn’t get sick due to water-related diseases. This might be due to the cold weather conditions of the area that causes restricted dispersal of disease transmitting microorganisms or vectors. The better experience observed in the area was that households had visited health centers during illness. Only 40.19% of the study population had visited health extension workers (HEWs), which is lower than the country’s health extension coverage. In addition, HEWs visited household workers infrequently. Reasons for this need further investigation.

Community participation in educational awareness activities in surrounding and/or the town sanitation and hygiene were very low with 18.42%. The quality of latrines was not observed, but 97.61% of the population had access to latrine facilities. The present investigator recommended further studies on the quality of latrines in the town.

A study undertaken in Ethiopia stated that about 36% of the population practiced open-defecation [13] whereas the present study indicated a lower percentage with3.59%. When the inaccessibility of water has occurred, people were forced to open defecation [8]. Globally, open defecation declined from time to time [4]. In middle-income countries, 35% didn’t have water for hand washing with water and soap [20]. The current study shows a lower percentage of 10.53%. Adequate water supply, good sanitation facilities, and proper hygiene practices improve the lives of the community [67]. The scarcity of water is associated with reduced sanitation facilities.

## 5. Conclusion

This study provides data on access, usage, and practice of water sources among urban households in northwest Ethiopia. It provides valuable insights into the access to safe water and consequent demographic, socioeconomic conditions such as income, distance, and family size, sanitation, and hygiene perceptions of households that can be associated with access to improved water sources.

The major findings suggest that 78.95% of households used improved and 21.05% of households used unimproved water sources. Based on the reported evidence, the study suggests that as in most developing countries in Ethiopia, specifically in the study area, the scarcity of water, especially from improved sources was very severe. Particularly the town population is under the problem of water scarcity. Increasing demand from a population for safe and quality water forces the local governments to increase their supply. However, due to the lower supply of pure water to households, people put in force for using water from unimproved sources, which have a possibility to contaminate with infectious microorganisms and cause water-borne diseases. Even if the presence of adequate drinking water is vital for humans, only 74.40% of the population consumes safe water and the rest is below the standard.

The cause of unsafe water quality for a population of 52.19% is due to the water-containing material, indoor practices that are due to water shortages. Animal and human wastes are the second cause of water quality deterioration. It is better to protect water sources from any contamination and use water treatment measures, when the water is stored for a longtime.

In conclusion, the uses of drinking water from improved sources were determined by different demographic, socio-economic, sanitation, and hygiene-related factors. This study suggests an association of the sources of drinking water with the age of the household head, the educational level of the household head, the source of income, monthly income, facilities observed, cleanness status of the surrounding, water scarcity, and family size. These factors significantly determine the sources of water, from improved or unimproved sources, while the availability of toilet facilities, household member type, and sex of the household head were not significant. Thus, older headed households were closely related to the availability of improved sources of drinking water. The educational status of the household head significantly determined the type of water source to be used. The type of source of income associated with the type of water source to be used in the households (i.e., 86.2%; 5.31% and 1.82%; for government employer, merchant, and self-employed), respectively. It is recommended that the local, regional, and national governments and other supporting organizations shall improve the accessibility and adequacy of drinking water from improved sources through short and long time plan for the well-being of the community in the area.

In the long-run Health Extension Workers (HEWs) shall be given attention for the improvement of the community sanitation and hygiene practices and give awareness. This might reduce the practice of open defecation.

## 6. Limitations

This study was conducted with the data collected from the households’ perceptions about the source of water use, water quality, and sanitation and hygiene practices in the area. Thus, the current study didn’t undertake verifiable water quality measures.

## Abbreviations

CI: Confidence Interval
CSA: Central Statistics Agency
DTT: Debre Tabor Town
EDHS: Ethiopian Demographic and Health Survey
HEW: Health Extension Worker
ICF: International Children’s Fund
JMP: Joint Monitoring Program
l/p/d: liters per person per day
OR: Odds Ratio
SDW: Sources of Drinking Water
SNNP: Southern Nations and Nationalities of Peoples
UNICEF: United Nation International Children Fund
WHO: World Health Organization
